# Insanity, Expert Medical Testimony and the Courts

**Published:** 1895-09

**Authors:** 


### INSANITY, EXPERT MEDICAL TESTIMONY AND THE COURTS.

We call attention to the report of the Allegheny County Medical Society in this issue, in which the medico-legal relations of the insane are discussed by well known representatives of medicine and law. Many late cases of homicide, in which the insanity of the criminal has been alleged as defense, make this report at this time especially appropriate and interesting, and we commend it to the careful thought of our readers.

It would be too much to expect a unanimity of medical opinion upon all the questions raised in these papers. One thing, however, stands out very prominent, and it is in accordance with every-day experience in the court-room, that so-called medical expert testimony is often absoluely worthless, serving only to show how doctors, the best of them, may disagree, and to confuse the jury rather than assist them in their conclusions. But doctors are no more infallible than the remainder of mankind, and just so long as the present method of choosing expert witnesses prevails, just so long will the doctors, *nolens volens*, be influenced in their opinions, and just so long will expert testimony be held in small esteem by the courts.

Another point brought out in these papers is the necessity for a revision of the legal idea of insanity in accordance with our advanced knowledge of diseased mental conditions. The legal criterion of insanity, that a man shall not know the difference between right and wrong, is very inadequate from a medical standpoint, and its rigid enforcement has already sent criminals to the gallows who should have been patients in our asylums. In this connection we take pleasure in referring the reader to an excellent article by Dr. J. B. Ransom, Physician to the Clinton (N. Y.) Prison ("Shall

Insane Criminals be Imprisoned or Put to Death?"), *Medical Record*, July 13. The author believes that our present methods of apprehending and trying criminals are much at fault, especially with reference to their mental condition. The New York *Law Journal* is now in favor of making some allowance for those offenders who may be "partially insane," and is willing to modify the old test, a knowledge between right and wrong.

We think the method of having a board of competent physicians to act in an advisory capacity with the court would remove many of the evils attending the present employment of expert witnesses; it would further the ends of justice, and temper this justice with mercy.

---

THE August issue of the Buffalo *Medical Journal* is the jubilee number, beginning the second half-century of its existence. Besides some excellent medical and surgical articles, it contains a full history of the *Medical Journal*, from its organization in 1845, by the elder Austin Flint, through all its administrations down to the present time. The same article, which is by the present able editor, Dr. Wm. Warren Potter, deals with other medical matters in the city of Buffalo for the same period. We congratulate our esteemed exchange upon its past history, and wish it a long and prosperous future.

---

THE *Woman's Medical Journal* (Toledo, Ohio) for August is of unusual interest, particularly for the female medical world. This number begins a series of biographical sketches of prominent female physicians, presenting sketches of Dr. Marie Zakrzewska of Boston, Dr. Eliza Burnside of Philadelphia, and Dr. May Spink of Indianapolis. The *Woman's Journal* is neatly gotten up and well edited.

